# The AHS-R: A holistic thinking measure with expanded theoretical domains and improved score reliability

**DOI:** 10.1371/journal.pone.0353378

**Published:** 2026-07-15

**Authors:** Ezgi Aytürk, Nilüfer Göktaş, Nagihan Özman, Emre Kayatepe, Rüya Su Şencan, S. Adil Sarıbay

**Affiliations:** 1 Department of Psychology, Koç University, Istanbul, Türkiye; 2 Department of Psychology, University of Southern California, Los Angeles, California, United States of America; 3 Department of Psychology, Özyeğin University, Istanbul, Türkiye; 4 Department of Psychology, University of Limerick, Limerick, Munster, Ireland; 5 Department of Psychology, KU Leuven, Leuven, Belgium; 6 Department of Psychology, Kadir Has University, Istanbul, Türkiye; Gelisim University: Istanbul Gelisim Universitesi, TÜRKIYE

## Abstract

Analytic and holistic thinking represent distinct cognitive styles. The existing Analysis-Holism Scale (AHS) is the most widely used measure of holistic thinking but has faced psychometric challenges and its validation has been largely restricted to a limited cultural context. The purpose of this study was to develop a refined measurement instrument, the AHS-R (Analysis-Holism Revised Scale), that expands the theoretical representation of the holistic cognition domains by differentiating between the Midway (preference for compromise) and Contradiction (tolerance for contradiction) dimensions and offers improved psychometric properties for use in broader populations. Using Turkish samples, Study 1 (*N* = 346) confirmed the feasibility of new items developed to more precisely and reliably represent Midway and Contradiction. Study 2 (*N* = 631) applied item response theory (IRT) and hierarchical factor analytic models on the finalized scale with four dimensions (Causality, Midway, Contradiction, and Attention). The original Perception of Change subscale was removed due to its negative factor loading on the higher-order holistic thinking factor. IRT analysis showed that measurement properties were optimized by collapsing the original 7-point rating scale to a 4-point scale. A structural equation model confirmed the presence of a dominant general holistic thinking factor and provided preliminary evidence of construct validity, showing that the unique variance of all four dimensions was significantly and positively related to increased adherence to preventive health behaviors during COVID-19. The newly developed AHS-R is a robust instrument for measuring holistic thinking that addresses key limitations of the original AHS by expanding its theoretical domains, notably through the clear distinction between the Midway and Contradiction subscales, and enhancing reliability. These improvements allow for more precise measurement, facilitating interpretation of scores across different levels of analysis. Ultimately, AHS-R supports further cross-cultural research into the relationship between cognitive style and behavior, such as health engagement.

## Introduction

Culture is believed to shape individuals’ perception, attention, memory, judgment, and decision-making processes and cognitive styles through socialization, social institutions (e.g., education, family), and social rituals [1–4]. Concurrently, individuals’ cognitive styles and behaviors contribute to culture through language, art, and tradition [4]. Nisbett et al. [5] have argued that the systems of thought and philosophy of ancient Chinese and Greek civilizations had a major influence on modern Eastern and Western societies, respectively. They referred to the Eastern thinking style as *holistic* and the Western thinking style as *analytic*. Holistic and analytic thinkers differ in the perception of part-whole relations, attention to object-context relations, categorization preferences, causal inferences, expectation of change, attitudes towards opposing views, and contradictory situations [5–6].

Analytical thinking is the tendency to see the world as composed of independent entities, to decontextualize the main object (or focal agent) of perception, and to focus on its individuating features when making categorizations and causal inferences. Compared to holistic thinkers, analytic thinkers pay more attention to the individual elements and details rather than the whole when making evaluations [3,4,7]. Analytic thinkers are inclined to predict future stability and perceive reality as relatively fixed and stable. According to Aristotelian logic (an essential part of Western thinking), contradictory views cannot be simultaneously true, and there is no middle ground [8]. In accordance with this view, analytic thinking involves resolving contradictions rather than accepting them by choosing one option (e.g., as correct or valid) over others [5,8,9].

In contrast, holistic thinking is the tendency to view the world as made up of intertwined and interconnected elements, to evaluate individual parts through examination of their relationship with the whole, and to assess objects, subjects, and events within their contexts when making causal attributions**.** Additionally, holistic thinkers expect continuous change from the future, and this dynamic nature of the world can create different realities that contradict each other [3,8]. Holistic thinkers also tend to accept these contradictions. Consistent with the emphasis on group harmony in ancient Chinese society, holistic thinkers believe that opposing views can exist in harmony and seek a middle ground in conflicting situations [5].

Holistic thinking style can be valuable in comprehending the modern world’s complicated problems (e.g., climate crisis, pandemics) that require realizing and perceiving complex causal relationships between events and underlying causes. In line with the principles of holistic thinking, assessing a phenomenon in its social or ecological context provides the opportunity to evaluate the problems from a broader perspective and to arrive at more balanced judgments and comprehensive explanations about the world. An individual who adopts the idea that a person is a part of a bigger whole (e.g., groups, society, the planet) and that everything in the world is interconnected would better comprehend that each individual is part of the change and solution. For instance, individuals with a stronger holistic thinking style intended to make more donations to nonprofit organizations for COVID–19 purposes, mediated by their belief that every small movement can cause a change [10]; and had stronger intentions to behave pro-environmentally, mediated by greater awareness of environmental risk and feelings of affinity toward nature [11]. Holistic thinkers attribute more responsibility to casualties when the outcomes are more complex (e.g., consisting of both positive and negative consequences) as compared to analytic thinkers [12]. The influence of holistic thinking may extend beyond pro-social and environmental domains into the realm of preventive health behaviors. Theoretically, holistic thinkers may be better equipped to comprehend the complex causal relationships inherent in global health crises, such as pandemics, by evaluating phenomena within their broader social and ecological contexts. This perspective fosters the belief that an individual is part of a larger whole—such as a society or planet—and that their actions are interconnected with collective well-being. Empirical evidence supports this link. For instance, research utilizing the Theory of Planned Behavior [13] has shown that holistic dimensions like “belief in connection” and “acceptance of change” significantly predict health-promoting lifestyles [14]. Furthermore, holistic thinking has been found to reduce health and safety risk-taking intentions by enhancing an individual’s awareness of potential consequences and their interconnectedness with the social environment [15]. Therefore, it is important to understand individual differences as well as cultural differences in the tendency to think holistically.

The Analysis-Holism Scale (AHS) [6] is the most widely used holistic-analytic thinking measure in literature. Following Nisbett et al. [5], AHS was developed to capture the four dimensions underlying holistic thinking: (1) *Causality* represents recognizing complex causal relations between different events and objects; (2) *Attitude toward contradictions* represents willingness to accept contradictory opinions and search for a middle ground for compromise; (3) *Perception of change* represents the expectation of change over time; and (4) *Locus of attention* represents the tendency to pay attention to the background and the whole versus just the figure and individual parts when making evaluations or judgments. For brevity, we will refer to these as *Causality*, *Contradiction*, *Change*, and *Attention*, respectively.

Two substantive critical issues have been identified regarding the construct validity and measurement model of holistic thinking style using AHS [6, 16–18]. Firstly, the factorial validity of the multidimensional holism construct has not been explicitly addressed. The AHS is often evaluated using correlated-factors confirmatory factor analysis (CFA) models. However, these models are not true measurement models because they do not include a common, target dimension (holism) being measured [19]. Consequently, these correlated-factors CFAs fail to formally evaluate the patterns of inter-factor correlations (which are inconsistent across studies). Crucially, they also do not provide a basis for how the total AHS score or subscale scores should be computed and interpreted, leaving the construct’s structure ambiguous. The *Change* subscale, in particular, has shown inconsistent factor loadings and reliability across various studies, suggesting potential instability in its measurement properties. This may be partly because the subscale is composed entirely of reverse-worded items except for one, a factor often linked to confusion and resulting measurement error (e.g., [20–23]).

Second, there is a conceptual issue in the *Contradiction* subscale. Specifically, items in the *Contradiction* subscale primarily emphasize a middle way approach (e.g., “It is more desirable to take the middle ground than go to extremes.”). This specific emphasis appears to stem from the common characterization of Easterners’ perception of contradictions [24], rather than measuring the tendency to embrace contradiction per se. This “middle way” focus might introduce a confounding variable. If one considers conservative disposition to maintain the social order and to resist societal changes [25], this could lead to positive associations between holistic thinking score (as measured by this subscale) and conservative ideologies and attitudes. This confounding, in turn, might distort the observed relationship between holistic thinking and some of the important sociopolitical attitudes such as pro-environmental attitudes. Supporting this expectation, Şencan [26] found that the original subscale focusing on *Middle way approach* was consistently related to social conservatism across two studies, whereas the newly created subscale for *Contradiction* was not.

Beyond the conceptual issues, the AHS also suffers from some psychometric inconsistencies and a lack of broad cultural validation. Psychometric inconsistencies include inconsistent item sets and reliability estimates. Specifically, no consensus has been reached on the measurement of the individual dimensions of AHS. Previous studies (e.g., [16–17]) proposed different sets of items for the subscales. Consequently, inconsistent patterns of internal consistency reliability (which were less than ideal at times), and apparent redundancy in items (correlated residuals) have not been addressed systematically across these studies. Also, previous studies did not examine the suitability of a seven-point response scale for AHS. This is a crucial omission, as modifying the response scale can remedy some psychometric problems [27]. S6 Appendix presents a summary of prior psychometric work on AHS, as well as the full pool of original and newly developed items evaluated across past and current studies.

Finally, the psychometric properties of AHS scores have been systematically evaluated in a limited range of cultural contexts. Despite the scale being introduced more than 15 years ago, we are aware of validation studies in only four non-English-speaking samples (Czech, South Korean, Mexican, and Spanish).

To address these significant psychometric and conceptual gaps, and to broaden the scale’s generalizability, evaluating and refining the AHS scores in the Turkish context is crucial. To begin with, data from Türkiye allows us to help expand psychological science’s database beyond WEIRD [28] cultures. Moreover, Türkiye offers a valuable testing ground for validating cognitive style measures because it serves as a unique geographic and cultural bridge between Eastern and Western influences. Moving beyond the traditional East-West binary, research in Türkiye reveals a distinctive psychological profile that blends autonomous and relational self-characteristics [29–31]. Central to this profile is the cultural logic of honor, a shared commodity where individual actions are perceived to have direct spillover effects on the reputation of one’s family and community [30]. In this context, health decisions, such as following pandemic protocols, are not merely individual choices but may be framed by interconnectedness and shared responsibility [32]. Validating and subsequently refining the instrument (Analysis-Holism Revised Scale; AHS-R) in Türkiye will provide a vital test case for the generalizability of the analytic-holistic dichotomy and enable more nuanced cross-cultural comparisons. This relational context also offers an opportunity to examine whether holistic cognitive style, which prioritizes social harmony and the recognition of complex causal relationships, can effectively predict adherence to collective preventive health behaviors.

Therefore, the present research pursues two major, interconnected goals that advance the measurement of holistic thinking, utilizing the opportunity afforded by the scale’s adaptation to the Turkish context. The first goal is the theoretical and structural refinement of holistic thinking construct and its measurement. This includes empirically testing the conceptually identified nuance between the Middle Way (*Midway*) and Attitude toward Contradiction (*Contradiction*) dimensions of holistic thinking, which is a crucial theoretical refinement not addressed in the original AHS; and improving measurement quality by evaluating the suitability of the seven-point rating scale and recommending necessary modifications.

The second goal is rigorous psychometric modeling and clarification of score interpretation. This is accomplished by testing the factorial validity of holistic thinking by fitting a second-order factor model. This strategy simultaneously tests for an overarching “holistic thinking” construct and quantifies the unique reliable information captured by the subscales, allowing us to explicitly discuss the implications for the use and interpretation of total versus subscale composite scores for the AHS. To illustrate the practical utility of these psychometric findings, we demonstrate how different uses of AHS scores lead to different results when predicting individuals’ self-reports of preventive behavior during the COVID-19 pandemic. Finally, these analyses contribute to the ongoing discussion on construct validity by underlining the importance of avoiding any cross-level generalizations on holistic thinking based on research that relies on individual differences measures. The data, code, and study materials for these studies can be found at https://osf.io/7vmnj/?view_only=d4b2de9a92314706bb06ea373f598d15.

### Study 1

Study 1 aimed to develop new items to capture the theoretical distinction between the Middle Way approach and Attitude towards Contradiction, and improve the internal consistency reliability of the AHS subscales. The original AHS subscales demonstrated lower-than-desired reliability (Cronbach’s αs ranged from .56 to .71 in [6]). Given the relationship between measurement reliability and statistical power (e.g., [33]), even minor reliability improvements were deemed essential. A pilot study was conducted to address these issues. Participants were recruited from 20/12/2018–11/04/2019 for Study 1 and its pilot study.

### Pilot study

We constructed seven new items focusing on contradiction (e.g., “Everything in the universe may contain opposite characteristics”), hypothesizing it as a distinct dimension of holistic thinking. In the rest of the manuscript, we refer to this new subscale as *Contradiction* and the original subscale (i.e., termed “Attitude toward Contradiction” by Choi et al. [6]) as *Middle way Approach* (or *Midway*). Additionally, to improve existing subscale reliability, we developed two new items for each of the *Causality*, *Change*, and *Attention* subscales using a logical-content strategy modeled after the original items.

A sample of undergraduate students enrolled in introductory psychology courses at Boğaziçi University (*n* = 137) participated in the online pilot study in exchange for course credit. They were administered the 37 items (24 original AHS items and 13 new items) of analytic-holistic thinking in addition to several other measures in random order. Informed consent was obtained from all participants via an online interface, where they provided agreement by clicking a checkbox after reading the consent form. The study received ethics approval from the Social Sciences and Humanities Human Research Ethics Board at Boğaziçi University.

For *Causality*, one new item (“It is possible that there is a causal relationship between two events that seem unrelated at first sight”) with a strong item-rest correlation (*r* = .74) was retained. For *Change*, one new item (“A continuously increasing state is more likely to increase rather than to decrease”; reverse-scored) which was moderately correlated with the original items (*r* = .34) was retained. The two new items of *Attention* yielded a less clear picture but were retained for further analyses. Importantly, the new *Contradiction* subscale had a Cronbach’s α of .67, which, even if not particularly strong, is as high as one of the original subscales in this sample (i.e., *Change*, .69). All but one of the seven *Contradiction* items showed item-rest correlations above .25 (the weakest being *r* = .095; its removal increased α to .71). *Contradiction* scores correlated non-negligibly and positively with the original AHS subscale scores: *r* = .54, 95% CI [.41 − .65] with *Causality*; *r* = .43, 95% CI [.28 − .56] with *Attention*, and *r* = .41, 95% CI [.26 − .54] with *Midway*. The only exception was the relatively weak, though still positive, correlation with *Change*, *r* = .11, 95% CI [−.06 − .27]. Crucially, this pattern persisted in partial correlation analyses controlling for *Midway* scores, suggesting the newly created *Contradiction* scale is not redundant with *Midway*.

Following the pilot study, we opted to retain the newly developed *Contradiction* subscale, incorporating five new items. Additionally, four new items (one each for *Causality* and *Change*, and two for *Attention*) demonstrated promising potential to enhance the scale’s psychometric characteristics. Hence, the next step involved evaluating the psychometric properties of this refined 33-item version of the AHS, which we refer to as AHS-33.

## Method

### Participants

346 undergraduate students (*M*_age_ = 20.92, *SD* = 2.29; 199 females) enrolled in introductory psychology courses at Boğaziçi University participated in the study in return for course credit. Twelve incomplete participations were excluded.

### Materials and procedure

The study received ethics approval from the Social Sciences and Humanities Human Research Ethics Board at Boğaziçi University. Informed consent was obtained from all participants via an online interface, where they provided agreement by clicking a checkbox after reading the consent form.

As part of a broader online study of cognitive style and political ideology [26], we administered the AHS-33 alongside several other measures.

The factor structure of AHS scores was evaluated with CFA using the *lavaan* package (version 0.6.15 [34]) in R (version 4.2.0 [35]). We compared the global fit of the CFA models and examined the model solutions. Adequate fit was indicated by a χ2/*df* ratio of ≤ 3 [36], Comparative Fit Index (CFI) values  ≥  0.90, and Root Mean Square Error of Approximation (RMSEA) values  <  0.08 [37]. Due to the multivariate non-normality of the data, we reported robust fit indices [38]. Our sample size meets the recommended minimum of 200 for CFA using maximum likelihood estimation [39]. The subject-to-item ratio was 9.6, which is slightly below the conventional rule-of-thumb criterion of 10 [40–41]. However, item communalities were high, which justifies the sample size.

## Results

The four correlated-factors CFA model of the 24-item AHS (which we refer to as AHS-24) that was originally proposed by Choi et al. [6] did not fit well to data (χ^2^/*df* = 3.1, CFI = .77, RMSEA = .084).

The second CFA was specified to reflect the new five-factor conceptualization of the holistic thinking style as measured by AHS-33. This model fit the data slightly better than the four-factor model (χ^2^/*df* = 2.6, CFI = .76, RMSEA = .072). For *Causality*, factor loadings ranged from .62 to .72 (*p*s < .001), and the new *Causality* item (“It is possible that there is a causal relationship between two events that seem unrelated at first sight”) showed a large standardized loading of .70 (*SE* = .06, *p* < .001). Factor loadings of the *Midway* ranged from .39 to .81 (*p*s < .001), and those of the new *Contradiction* subscale ranged from .32 to .67 (*p*s < .001). Factor loadings for the *Change* subscale ranged from .23 to .67 (*p*s < .001); and the new *Change* item (“A continuously increasing state is more likely to increase rather than to decrease”) showed a loading of .41 (*SE* = .09, *p* < .001). For *Attention*, factor loadings of the original items ranged from .11 (*SE* = .07, *p* = .07) to .84 (*p*s < .001). Loadings of the two new items were divergent: one was negative at −.13 (*SE* = .06, *p* = .02; “A detail noticed afterwards can totally change our view of a phenomenon”, while the other was .49 (*SE* = .09, *p* < .001; “When a system is corrupted, it is more important to focus on the whole system rather than on the single broken part”). Modification indices suggested freely estimating the residual correlations between several pairs of item scores.

Factor scores from the new *Contradiction* subscale correlated strongly with those of *Causality* (*r* = .81, *SE* = .05, *p* < .001), and moderately with those of *Midway* (*r* = .39, *SE* = .06, *p* < .001), *Change* (*r* = .26, *SE* = .07, *p* < .001), and *Attention* (*r* = .33, *SE* = .06, *p* < .001). Internal consistencies of all subscales for which we developed a modified version, except for *Attention*, were improved in the five-factor model of AHS-33 scores. Cronbach`s α estimates were .86 (*Causality*), .74 (*Midway*), .67 (*Contradiction*), .69 (*Change*), and .66 (*Attention*).

## Discussion

In Study 1, we developed new items to include in AHS to reflect the theoretical distinction between *Contradiction* and *Middle way* approach and to improve the internal consistencies of the remaining subscales. Based on initial psychometric analyses, we decided to keep nine of the new items for further analysis. We called this version of the scale AHS-33. CFA results suggested that five dimensions may be adequate to reflect the factorial structure of holistic cognition.

The correlated residuals observed in Study 1’s CFA suggested item redundancy and necessitated a more rigorous test of dimensionality. Furthermore, Study 1 recruited only undergraduates, which limits the generalizability of the findings.

### Study 2

Study 2 was designed to address the limitations of Study 1 by conducting a more rigorous and comprehensive psychometric evaluation of the AHS-33. We recruited a large, demographically representative sample from Türkiye for the main data collection. Prior to psychometric analyses, we conducted cognitive interviews with a small subsample of this population to assess the clarity and meaning preservation of the AHS-33 items.

We then administered the refined AHS-33, and used CFA measurement models (second-order factor model) and IRT to test the overarching ‘holistic thinking’ construct and optimize the response scale, leading to an improved multidimensional scale (named AHS-R). Next, we examined the associations between participants’ scores on AHS-R and the degree to which they reported engaging in preventive behaviors during the COVID-19 pandemic as measured by the Preventive Behaviors Scale (PBS) [33]. However, experiential or intuitive thinking have been associated with vaccine hesitancy or engagement in pseudoscientific health practices when the role of rational reasoning is suppressed [42]. Importantly, these modes of thought are also considered holistic according to some theorists [43]. Consequently, exploring how specific facets of holistic thinking relate to compliance with public health measures, such as preventive health behaviors, provides a critical avenue for understanding these nuanced cognitive dynamics. The role of PBS scores in this study is twofold: first, PBS is chosen for validity purposes because both conceptually (holistic thinkers are more likely to engage in collective health behaviors with a tendency to think that the self and others are interconnected, that every small action matters and a tendency to pursue interpersonal harmony) [4,5] and empirically (e.g., [14]) PBS scores are expected to be positively associated with holistic thinking style to at least a moderate extent. Second, we used the PBS association to illustrate how validity evidence may be influenced by different scoring methods (observed versus factor scores) of the AHS, contrasting results from multiple regression and structural equation models (SEM). Participants were recruited from 25/10/2022–30/12/2022 for Study 2.

### Cognitive interviews

Interviews with 11 individuals (6 women; *M*_age_ = 40.6, *SD* = 13.1) assessed the clarity and meaning preservation of AHS-33 items post-adaptation and translation. Revisions were made to Item 14 (simplified sentence structure), Item 25 (rewritten for clarity), and Item 7 (improved comprehension). All other items were deemed comprehensible based on these interviews. This refined AHS-33 version was then used for data collection.

## Method

### Participants

The study recruited 750 participants via an independent research company. To ensure data quality, 100 participants who completed the survey in under 10 minutes, 17 who participated multiple times, and 2 with identical responses were excluded, resulting in a final sample of 631 participants. Sample demographics are presented in Table 1.

**Table 1:** Sample demographics.

	Overall(N = 631)
Age	
Mean (*SD*)	33.7 (10.1)
Median [Min, Max]	32.0 [17.0, 78.0]
Age Group	
18 - 24	123 (19.5%)
25 - 34	234 (37.1%)
35 - 44	188 (29.8%)
45 - 54	62 (9.8%)
55 - 64	17 (2.7%)
65 age and over 65 years old	5 (0.8%)
Under 18 age	2 (0.3%)
Gender	
Female	382 (60.5%)
Male	249 (39.5%)
Ethnicity	
Arab	3 (0.5%)
Armenian	1 (0.2%)
Balkan	6 (1.0%)
Caucasian	7 (1.1%)
Greek	1 (0.2%)
Kurdish	62 (9.8%)
Other	7 (1.1%)
Turkish	544 (86.2%)
Religion	
Atheist	18 (2.9%)
I believe in god but do not believe in a religion	45 (7.1%)
Muslim	562 (89.1%)
Other	6 (1.0%)
Political Ideology	
Apolitic	21 (3.3%)
Conservative democrat	96 (15.2%)
Islamist	85 (13.5%)
Kemalist	106 (16.8%)
Liberal	15 (2.4%)
Other	30 (4.8%)
Social democrat	156 (24.7%)
Socialist	49 (7.8%)
Turkish nationalist	73 (11.6%)
Education Level	
Bachelor’s degree	236 (37.4%)
College student	109 (17.3%)
Graduate student	24 (3.8%)
High school graduate	147 (23.3%)
Master’s/PhD. degree	33 (5.2%)
Primary school graduate	32 (5.1%)
Vocational school graduate	50 (7.9%)
Income Level	
Bad	158 (25.0%)
Good	109 (17.3%)
Not bad	317 (50.2%)
Very bad	34 (5.4%)
Very good	13 (2.1%)
Hometown Size	
Borough	17 (2.7%)
City	162 (25.7%)
Metropolis	420 (66.6%)
Small town	14 (2.2%)
Village	18 (2.9%)
Socio-Economic Status (SES)	
Mean (SD)	4.98 (1.83)
Median [Min, Max]	5.00 [1.00, 10.0]
Political Orientation (1 = Left, 7 = Right)	
Mean (SD)	4.32 (1.75)
Median [Min, Max]	4.00 [1.00, 7.00]
Missing	45 (7.1%)
Religiosity (1 = Not at all religious, 7 = Very religious)	
Mean (SD)	4.78 (1.07)
Median [Min, Max]	5.00 [2.00, 6.00]
Missing	145 (23.0%)

### Materials and procedure

The study received ethics approval from the Human Research Ethics Board at Kadir Has University. Informed consent was obtained from all participants via an online interface, where they provided agreement by clicking a checkbox after reading the consent form.

Participants completed the final version of AHS-33, the 4-item PBS [3 7] (α = .82), and a demographic form via an online platform. PBS was used to measure the preventive behaviors people engaged in during the COVID-19 pandemic. We adapted items to the COVID-19 context simply by replacing references to Middle East Respiratory Syndrome with coronavirus. We asked participants to consider their behavior during the peak periods of the pandemic in 2020 and 2021. Used items are, “*I wore a mask to reduce the risk of coronavirus.*,” “*I tried to avoid going to public places like restaurants or shopping malls.*,” “*I tried to avoid going to hospitals.*,” “*I tried to wash my hands more often or use hand sanitizer to prevent the risk of coronavirus*.”

The AHS-33 was administered first, followed by the PBS and the demographic form. All participants in the final sample passed the attention check and had complete data on the AHS-33.

## Results

Excepting the items of the *Change* subscale, respondents tended to strongly endorse (scored 6 or 7) the majority of the items. Initial item-rest correlations revealed several problematic items (Table in S1 Appendix). Notably, AHS-33 Item 32 correlated negatively with the other items measuring the *Attention* dimension, while three items from the *Change* subscale (Items 19, 24, and 25) and Item 31 showed negative overall item-rest correlations. This preliminary analysis highlighted potential issues with item coherence and the viability of the *Change* subscale.

### Initial CFAs and item selection

To select the best items for a multidimensional scale, we employed CFA with the goal of achieving a simple structure (each item loading strongly onto only one dimension) and a balanced number of items per theoretical dimension. CFA models were estimated using the weighted least square mean and variance adjusted (WLSMV) estimator, with robust standard errors calculated based on the full-weight matrix**.** Our sample size meets the minimum sample size requirements for CFAs with similar properties (e.g., [44]).

We first examined an *a priori* second-order factor model (Model 1) where a single second-order factor accounted for the covariances among the five hypothesized first-order factors: *Causality* (Items 1−7), *Midway* (Items 8−13), *Contradiction* (Items 14−18), *Change* (Items 19−25), and *Attention* (Items 26−33; see S1 Appendix). This initial model resulted in a poor fit (*χ2/df* = 9.5). The most critical issue was that the factor loading of the *Change* subscale on the second-order factor was negative (−0.49, *SE* = 0.05, *p* < .001). This finding, consistent with the poor item-rest correlations of the *Change* items, clearly indicated that this subscale was not measuring holistic thinking as defined by the general factor, justifying its removal from further analysis.

**Fig 1:**
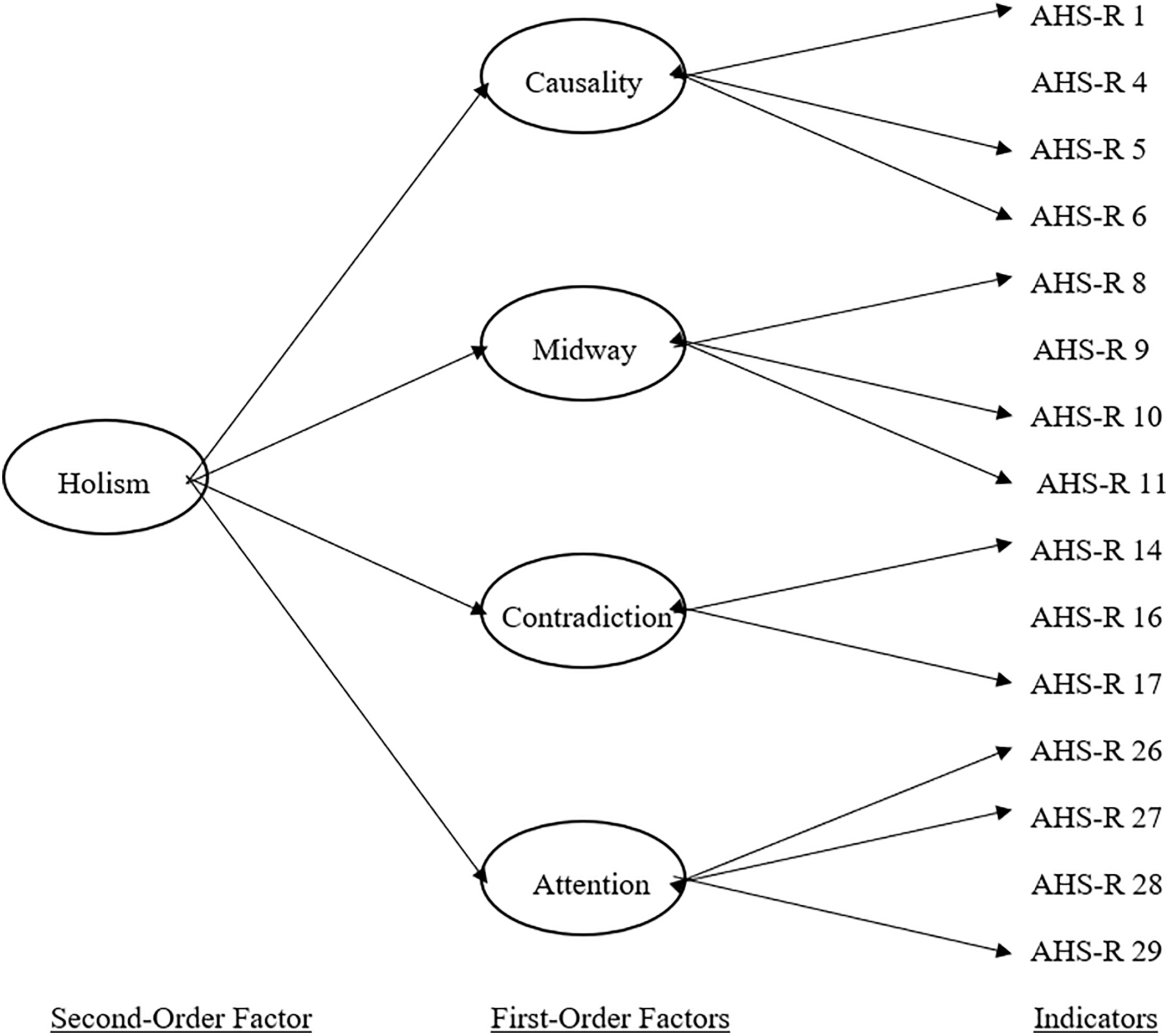
Conceptual second-order factor model with 15-item AHS-R.

We dropped the *Change* subscale and fit a new second-order factor model (Model 2) with the remaining four first-order factors. Although global fit improved, it remained poor (*χ*^*2*^*/df* = 7.4). We then proceeded through an iterative process of refinement (Models 3–8, detailed in S2 Appendix) based on modification indices, during which several items (32, 12, 31, 30, 18, 2, 15, 33) were sequentially removed to achieve a simple and well-fitting structure. Model 8, measuring *Causality* with six indicators, *Midway* with five, *Contradiction* with three, and *Attention* with four, fit well (*χ*^*2*^/*df* = 3.0, RMSEA = .08, CFI = 0.94). To have a balanced number of items in each scale, we retained the best four items for the *Causality* (Items 1,4,5, and 6) and *Midway* (8, 9, 10, 11) subscales each. This final model (Model 9) fit the data well (*χ*^*2*^/df = 2.8, RMSEA = .07, CFI = 0.95). Fig 1 shows the conceptual diagram of the final measurement model (Model 9). Both English and Turkish versions of the final set of items are presented in S7 Appendix.

### IRT analyses

**Fig 2:**
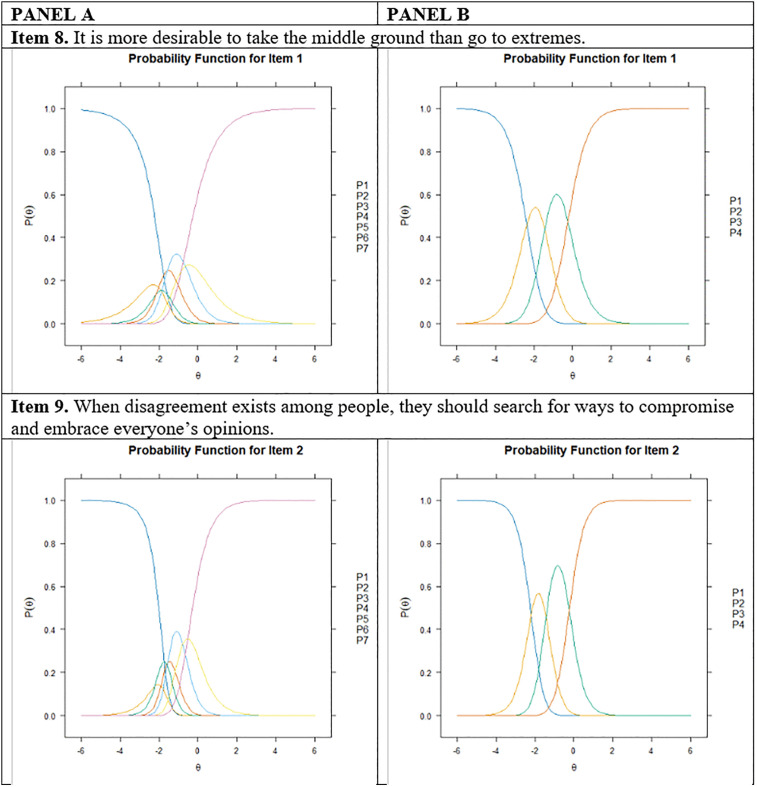
Category characteristic curves of two midway items.

The 16 items retained in CFA Model 9 were submitted to Item Response Theory (IRT) analyses to optimize the response scale. We initially fit unidimensional Partial Credit Models (PCMs) and Generalized Partial Credit Models (GPCMs) for each subscale separately. These unidimensional models were used to diagnose potential measurement problems, such as disordered thresholds, within the existing seven-point response format. Overall, GPCMs fit better than PCMs (S3 Appendix). However, the GPCMs showed that response category thresholds were either not ordered or had highly overlapping confidence intervals. Panel A in Fig 2 shows the category characteristic curves (CCCs) of AHS-33 Items 8 and 9 from the GPCM for illustration.

Based on the pattern of threshold estimates, we collapsed the second, third, and fourth response options and the fifth and sixth response options, resulting in a 4-point scale. Rerunning the unidimensional PCMs and GPCMs on the collapsed data showed ordered and well-separated thresholds, accompanied by substantially improved AIC and BIC values (S3 Appendix).

We then fit the second-order factor GPCM (a multidimensional IRT model) to the collapsed data (*C*₂(87) = 232.74, SRMSR = .09) which significantly outperformed the second-order factor PCM (Δ*X*²(28) = 211.37, *p* < .001). This multidimensional model served to confirm both the final four-factor structure and the improved measurement properties of the optimized four-point response scale. The model showed that items had a good fit to the model, and the effect size of any misfits (RMSEA) was low (S4 Appendix). Panel B in Fig 2 shows the new CCCs of AHS-33 Items 8 and 9 for illustration. We used the optimized four-point response scale for the rest of the analyses.

### The final measurement model

We confirmed the final structure by fitting the second-order factor CFA model (Model 9) to the 4-point collapsed data. This model fit well (*χ*^*2*^/*df* = 2.65, RMSEA = .74, CFI = 0.95). Standardized factor loadings were large, with first-order loadings greater than 0.67 and second-order loadings greater than 0.52 (Table 2). This provided evidence for the presence of a general holistic thinking factor encompassing at least four distinct subdomains.

**Table 2:** Second-order factor CFA solution after collapsing the response options.

	First-order factor loadings			
	Causality	Midway	Contradiction	Attention
AHS-R 1^*^	1.00 (0.83)			
AHS-R 4^*^	1.00 (0.83)			
AHS-R 5^*^	1.11 (0.91)			
AHS-R 6^*^	1.07 (0.88)			
AHS-R 8^*^		1.00 (0.83)		
AHS-R 9^*^		1.00 (0.83)		
AHS-R 10^*^		0.93 (0.77)		
AHS-R 11^*^		0.85 (0.71)		
AHS-R 14			1.00 (0.68)	
AHS-R 16			1.24 (0.83)	
AHS-R 17			1.23 (0.83)	
AHS-R 26^*^				1.00 (0.89)
AHS-R 27^*^				0.98 (0.88)
AHS-R 28^*^				0.75 (0.67)
AHS-R 29^*^				0.83 (0.74)
	Second-order factor loadings	Residual variances		
Causality	1.00 (0.87)	0.17 (0.25)		
Midway	0.93 (0.80)	0.25 (0.36)		
Contradiction	0.76 (0.80)	0.16 (0.35)		
Attention	0.64 (0.52)	0.59 (0.74)		

Completely standardized loadings are presented in parentheses.

* indicates items from the original AHS.

Table 3 presents the proportion of respondents in each of the four response options and the category threshold parameter estimates. In the rest of the analyses, we estimated the relative strength of the general factor versus the subdimensions in holistic thinking, specifically determining what portion of the reliable variance in each subscale is due to the general factor and what portion is due to the unique dimension they tap, and examining how this information is relevant when interpreting the relationship between holism subdimensions and external variables.

**Table 3:** Percentage (%) of item responses and threshold estimates.

Items	Response options				Threshold parameters		
	1	2	3	4	T1^a^	T2^b^	T3^c^
AHS-R 1^*^	1.0	7.4	23.9	67.7	−2.35	−1.38	−0.46
AHS-R 4^*^	1.0	10.5	35.2	53.4	−2.35	−1.20	−0.09
AHS-R 5^*^	0.8	7.3	34.2	57.7	−2.41	−1.40	−0.19
AHS-R 6^*^	1.3	10.0	32.6	56.1	−2.24	−1.21	−0.15
AHS-R 8^*^	1.9	10.1	33.8	54.2	−2.07	−1.17	−0.11
AHS-R 9^*^	1.9	8.6	33.9	55.6	−2.07	−1.26	−0.14
AHS-R 10^*^	2.4	10.1	34.2	53.2	−1.98	−1.15	−0.08
AHS-R 11^*^	4.1	14.9	36.3	44.7	−1.74	−0.88	0.13
AHS-R 14	2.4	30.9	39.5	27.3	−1.98	−0.43	0.61
AHS-R 16	1.6	20.0	46.3	32.2	−2.15	−0.79	0.46
AHS-R 17	1.0	20.6	43.4	35.0	−2.35	−0.79	0.38
AHS-R 26^*^	3.0	26.5	40.6	30.0	−1.88	−0.54	0.53
AHS-R 27^*^	3.3	28.4	38.2	30.1	−1.83	−0.48	0.52
AHS-R 28^*^	8.9	26.6	33.6	30.9	−1.35	−0.37	0.50
AHS-R 29^*^	4.9	30.9	37.2	26.9	−1.65	−0.36	0.61

^a^T1 = Threshold between response options 1 and 2.

^b^T2 = Threshold between response options 2 and 3.

^c^T3 = Threshold between response options 3 and 4.

* indicates items from the original AHS.

### Reliability decomposition and construct validity

We calculated McDonald`s ω for the AHS-R subscale scores, which estimates the reliability attributable to both the general holistic thinking factor and the specific dimensions. These values were large for all subscales: .93, .87, .83, and .88 for *Causality*, *Midway*, *Contradiction*, and *Attention*, respectively. However, ω-Hierarchical Subscale (ω-HS) values, which estimate the reliability of the subscale scores when the general factor is controlled for [45] (See S5 Appendix), were substantially lower. This was particularly evident for the *Causality* (.23), *Midway* (.32), and *Contradiction* (.30) subscales, indicating that these dimensions carry only a small amount of reliable unique information beyond the general factor. The ω-HS of *Attention* was notably larger (.64), implying its relative independence from the other subdimensions of holistic thinking.

**Fig 3:**
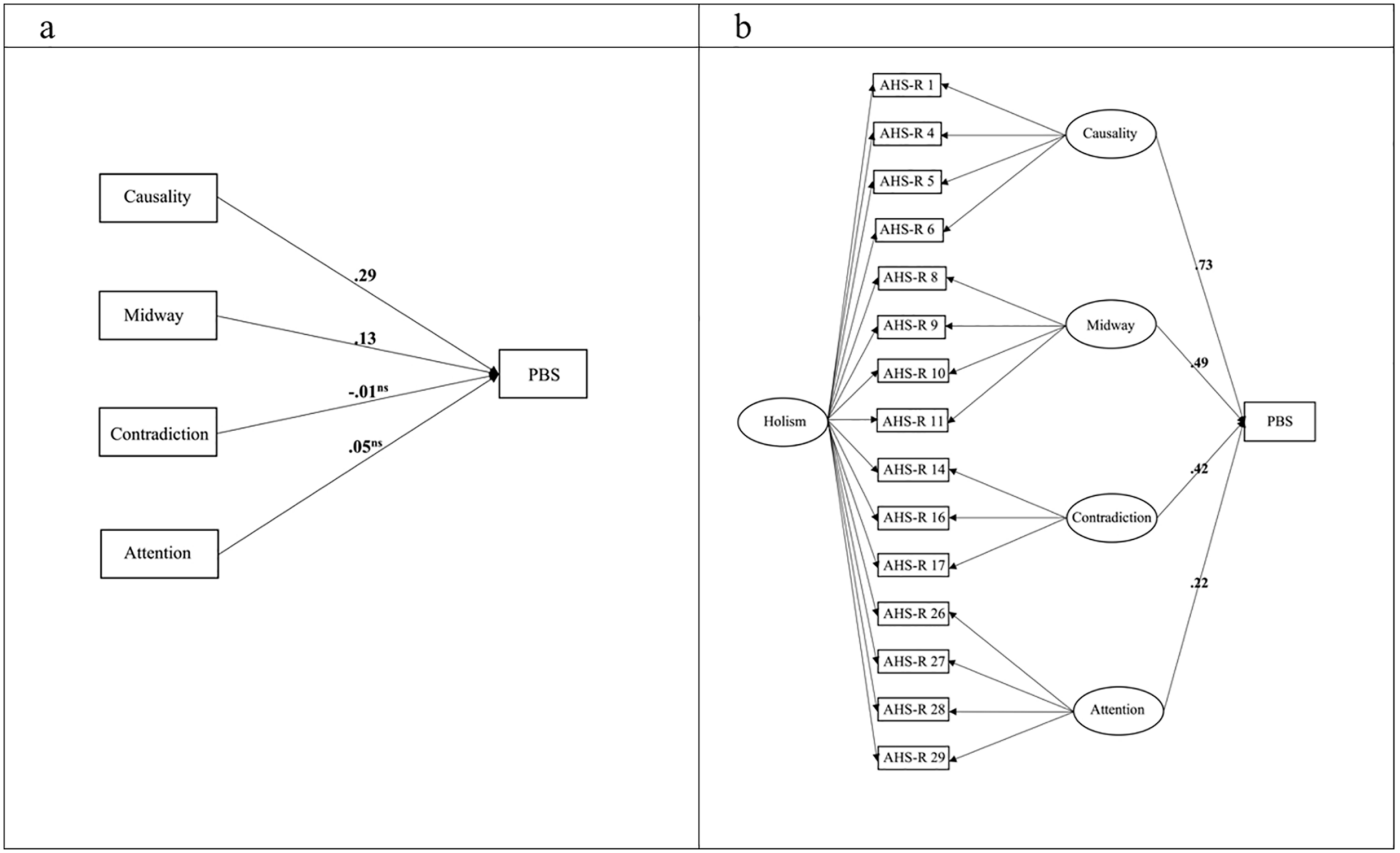
SEM and multiple regression in predicting preventive behaviors during theCOVID-19 pandemic.

We compared two approaches to predicting individuals’ self-reported engagement in preventive behaviors (PBS scores) during the COVID-19 pandemic. First, a multiple regression model using the observed (raw) AHS-R subscale scores (Fig 3a) showed that only *Causality* (*β = .29, SE = .05, p < .001*), and *Midway* (*β = .13, SE = .05, p < .01*) were significantly related to the PBS scores (*F*(4,626) = 28.5, *p* < .001, *R*^*2*^ = .15). Second, when the general factor was statistically partialled out from the subscale scores using a structural equation model (SEM) (Fig 3b), a much different pattern emerged. In the SEM, all four subdimensions (*Causality* (*β* =.73, 95% *CI* = [.64, .83], *p* < .001), *Midway* (β =.49, 95% *CI* = [.40, .58], *p* < .001), *Contradiction* (*β* =.42, 95% *CI* = [.32, .53], *p* < .001), and *Attention* (*β* =.22, 95% *CI* = [.15, .30], *p* < .001)) were significantly related to PBS scores (*χ*^*2*^/*df* = 2.5, RMSEA = .05, CFI = 0.99).

## Discussion

Drawing on the findings from Study 1, the goal of Study 2 was to tailor an improved measurement instrument for holistic thinking using contemporary statistical measurement models, moving beyond the correlated factors model employed in the original AHS study [6] and subsequent research (e.g., [17]). Measurement models provide justification for using total scores by formally testing for the presence of a general factor that organizes individual dimensions [39]. Our second-order factor model confirmed this structure, but it clearly showed that the *Change* dimension diverged from the rest of the construct. While it remains unclear whether this is due to *Change* representing a distinct factor or being contaminated by the reverse-wording effect, we recommend that researchers currently refrain from interpreting *Change* scores as part of the holistic thinking style measured by AHS. Future research should test the feasibility of *Change* as a subdimension of holism with a set of new, ideally forward-worded, items. Furthermore, using IRT, we determined that individual differences in holistic thinking style are best captured with a four-point response scale, as more options appeared to cause data-sparseness, which has important implications for statistical inference [27].

With the removal of the *Change* dimension and other minor modifications, the final second-order factor model demonstrated an acceptable fit, supporting the presence of a strong higher-order dimension within the AHS structure. The reliability decomposition analysis and the subsequent construct validity tests illustrated the implications of this structure. *Attention* stood out as a distinct subscale capturing the greatest amount of unique reliable information above and beyond the general factor, while the remaining subscales captured only minimal unique variance. This distinction was illustrated by the SEM and multiple regression models predicting self-reported preventive behaviors during the COVID-19 pandemic. Specifically, the standardized regression coefficients of all four dimensions were substantially larger when different sources of reliability (general vs. specific) were accounted for in the SEM compared to the multiple regression analysis using observed scores. Given these findings, we recommend that researchers opt for latent variable modeling techniques like SEM if they are interested in the unique effect of one or more dimensions of holism on external variables. On the other hand, given the strong general factor, total AHS-R score appears to be a reliable measure of the overall multidimensional holism construct.

## General discussion

This research contributes to the long-standing theoretical discussion regarding how culture shapes fundamental cognitive styles, specifically addressing the analytic-holistic dichotomy proposed by Nisbett and colleagues [5]. Holistic thinking, characterized by context-dependent attention, dialectical reasoning, and expectations of change, is essential for navigating complex, modern challenges, as demonstrated by its link to collective preventive behaviors during a pandemic. Given the importance of accurately measuring this construct for cross-cultural research and understanding social phenomena, our study aimed to address significant psychometric and conceptual limitations in the most widely used instrument, the Analysis-Holism Scale (AHS) [6]. We specifically focused on the AHS given its established popularity, despite the recent development of alternative measures like the Holistic Cognition Scale (HCS) [40], which itself faces similar conceptual issues, such as the conflation of the “middle way” approach and tolerance for contradiction.

With a thorough examination of the item response patterns from the Turkish version of AHS and the modified versions that we proposed, we developed the four-dimensional AHS-R with a four-point response scale. AHS-R excludes the *Change* dimension, scores on which did not align with the theoretical expectations.

### Theoretical implications

Our findings offer several significant theoretical implications regarding the structure of holistic cognition. The removal of the *Change* subscale due to its negative loading is a critical theoretical and methodological finding. Theoretically, the concept of *Change*, or the perception of the world as dynamic and fluid, is one of the core tenets of holistic thought. The failure of the original items to load onto the general factor raises concerns about their cross-cultural validity or their fundamental conceptualization. While the divergence may be due to the reverse-wording effect, a known contributor to measurement error (e.g., [20,22]), this subscale’s failure warrants a theoretical re-evaluation of how dynamic change is experienced and measured outside of its original conceptual context.

Supporting the theoretical importance of the *Change* dimension despite its psychometric failure in AHS, a rich body of cross-cultural research has documented robust cultural differences in change perception using behavioral and judgment-based paradigms. For instance, Ji et al. (2001) showed that Chinese participants were significantly more likely than Americans to predict reversals in ongoing trends, whether in social scenarios or graphed time-series data, and to anticipate nonlinear developments in events that were currently moving in a particular direction. Crucially, this work operationalized change perception through prediction tasks, covariation detection, and graph extrapolation, none of which rely on explicit belief statements about the nature of change, such as those used in the AHS. The consistency of cultural differences across these diverse behavioral measures suggests that holistic change perception is a psychologically real phenomenon, but one that may be particularly difficult to capture through the kind of reverse-worded Likert items employed in the original AHS *Change* subscale. Indeed, asking participants to disagree with statements like “If an event is moving toward a certain direction, it will continue to move toward that direction.” may introduce acquiescence and confusion artifacts that obscure genuine individual differences in the underlying construct. The items developed by Ji and colleagues, including judgments about the probability of trend reversals or the likelihood that an extreme state will persist, offer a promising template for developing new, performance- or scenario-based items that could reestablish *Change* as a viable and construct-valid dimension of holistic thinking in future iterations of the AHS-R.

Our scale refinement also involved splitting the original *Contradiction* dimension into two distinct constructs: attitude towards *Contradiction* and the *Middle Way* approach. This is not merely a statistical improvement but a crucial theoretical refinement. The middle way approach reflects a process-oriented cognitive strategy of actively seeking a synthesis or compromise when faced with two opposing sides. In contrast, *Contradiction* represents a more passive acceptance and tolerance of contradictions in the world. By treating these as separate dimensions in AHS-R, we gain theoretical precision, enabling researchers to distinguish between the motivation to reconcile and the ability to tolerate conflict, a nuance confounded in earlier measures.

Finally, the finding of a strong second-order general factor confirms the theoretical assertion that the various dimensions of holistic thinking (*Causality*, *Midway*, *Contradiction*, and *Attention*) are not merely correlated, but are manifestations of a single, underlying global construct: the overarching holistic thinking style. This strong general factor suggests that individuals tend to be consistently holistic (or non-holistic) across these different cognitive facets. The subsequent reliability decomposition provided insight into the structure of this general factor. The subscales *Causality***,** *Midway***,** and *Contradiction* are highly saturated by the general factor, meaning they are highly interconnected, at least statistically. In contrast, *Attention* stood out as a distinct subscale capturing considerable unique information above and beyond the general factor. This significant statistical distinction highlights that *Attention* is the most independent subscale, which, given its foundational perceptual nature, provides a basis for the hypothesis that it may represent a more fundamental, lower-level cognitive process that precedes the higher-order dialectical reasoning captured by *Causality*, *Midway*, and *Contradiction*.

### Methodological implications

The pattern of internal consistency estimates, particularly the low ω-HS values for *Causality*, *Midway*, and *Contradiction*, demonstrated that the observed subscale scores (except for that of *Attention*) are not sufficiently distinct from the general factor to be reliably interpreted in isolation. This phenomenon was illustrated in our prediction of self-reported preventive behaviors during the COVID-19 pandemic, where the latent variable modeling approach (SEM) revealed a more theoretically meaningful pattern of relations compared to the traditional multiple regression analysis using observed scores. Given these findings, we provide two primary methodological recommendations. First, researchers interested in testing the unique effect of one or more dimensions of holism on external variables must opt for latent variable modeling techniques like SEM, as this approach effectively controls for the general holistic thinking factor, isolating the specific subdimension effect. Second, conversely, given the presence of a strong general factor, the total AHS-R score appears to be a robust and reliable measure of the overall multidimensional holism construct and is suitable for use when examining global holistic thinking tendencies.

### Limitations

Despite the significant psychometric improvements offered by the AHS-R, our study has several limitations that should be addressed by future research. First, our investigation was limited to a single cultural context (Türkiye). While this context is theoretically interesting due to its position as a bridge between Eastern and Western cultures, the generalizability of the refined AHS-R structure and its measurement properties must be established across other cultural groups, particularly those where holistic thinking is traditionally stronger (e.g., Eastern cultures). Second, a key finding of our research, the mandatory exclusion of the *Change* subscale, highlights an inherent design flaw in the original scale, specifically the potential contamination from the reverse-wording effect. While we recommend developing new, forward-worded items to reestablish this dimension’s viability, the current AHS-R does not include a robust measure of the perception of change over time, which is a theoretically vital component of holistic thinking. Finally, as a cross-sectional study, our data cannot address the temporal stability of the AHS-R factors or the causal dynamics between holistic thinking dimensions and external outcomes like preventive behaviors. Future studies utilizing longitudinal designs would be necessary to establish the temporal stability and predictive validity of the AHS-R scores.

### A cautionary note on the cross-level generalization of findings on the individual differences in holistic thinking style

The development of AHS, as well as the newly developed Holistic Cognition Scale are claimed to improve our understanding of the holistic thinking style at the individual level (as opposed to the cultural level). For example, Lux et al. [47, p.10] stated that: “The HCS thereby creates an opportunity to shift gears in cross-cultural research by examining the cognitive schema that people use to engage the world, studying *how* they think at the individual level, instead of *what* they think at various levels of aggregate abstraction.” Although psychometric studies on these scales are based on data that are indeed provided by groups of individual participants, the findings in the current study, as well as those in Choi et al. [6], Martin-Fernandez et al. [17], and Lux et al. [46] are not relevant at the individual level. Relying on covariance structures of interindividual variances on item responses, the findings (e.g., factorial structures, reliabilities, and correlations) are interpretable at the aggregate (population) level from which the respective samples in these studies are coming from. Until it is established that the inter-individual measurement models presented in these studies are also obtained at the level of the individuals (i.e., intra-individual measurement models), that is, until the condition of homology is demonstrated across these levels (e.g., [47]), researchers should avoid making any top-down (aggregate to individual) generalizations regarding the psychological structure or process of holistic thinking style.

Having said that, AHS-R and other measures of individual differences on holistic thinking may be used to compare groups on the equivalence of the structure of holistic thinking (using measurement noninvariance testing, for example). Future research may focus on if and how (sub-)groups differ on the cognitive schemata (i.e., measurement structure of the analysis-holistic thinking) they use through which they perceive their worlds. For example, how well the dimensions of holistic thinking identified in the present and previous studies account for the inter-individual differences among homogeneous subgroups within a population or whether *qualitatively* different structures would better account for the individual differences within particular groups could be examined. Studies identifying structures of inter*individual* variation in increasingly homogeneous subgroups can help generate new hypotheses about the structure and process of holistic thinking at the *individual* level.

## Conclusion

With the development of the AHS-R, we have provided a more robust and theoretically precise measurement tool for holistic thinking. This includes the conceptual splitting of Contradiction and the establishment of a clear measurement model, offering strong evidence for the role of a dominant general factor in organizing holistic thought. Future research may evaluate the robustness of the findings and validity of AHS-R scores with new samples using a larger selection of criteria.

## Supporting information

S1 Appendix Percentage of item responses and item-rest correlations. (DOCX)

S2 Appendix Initial CFAs and item selection. (DOCX)

S3 Appendix Global goodness-of-fit of the unidimensional IRT models before and after collapsing. (DOCX)

S4 Appendix Item fit of the second-order factor GPCM with 4-point response scale. (DOCX)

S5 Appendix The bifactor model from which the hierarchical omega coefficients were estimated. (DOCX)

S6 Appendix Summary of prior psychometric work on the AHS. (DOCX)

S7 Appendix Turkish and English versions of the AHS-R. (DOCX)
