# Supplementary material for: The AHS-R: A holistic thinking measure with expanded theoretical domains and improved score reliability
Source: PLoS One. 2026 Jul 15;21(7):e0353378. doi: 10.1371/journal.pone.0353378 (PMC13372108; doi:10.1371/journal.pone.0353378)
Supplement: S1 Appendix — (DOCX) [file pone.0353378.s001.docx]

S1 Table. Percentage (%) of item responses and item-rest correlations

|  | Response options | | | | | | | Item-rest correlations | |
| --- | --- | --- | --- | --- | --- | --- | --- | --- | --- |
| Item | 1 | 2 | 3 | 4 | 5 | 6 | 7 | Subscale | Overall |
| AHS-R 1* | 1.0 | 0.6 | 2.1 | 4.8 | 9.5 | 14.4 | 67.7 | .76 | .61 |
| AHS-R 2* | 3.6 | 2.5 | 3.8 | 10.8 | 10.6 | 15.7 | 52.9 | .62 | .49 |
| AHS-R 3* | 1.0 | 1.4 | 2.1 | 8.6 | 14.6 | 19.2 | 53.2 | .74 | .58 |
| AHS-R 4* | 1.0 | 1.3 | 3.0 | 6.2 | 16.0 | 19.2 | 53.4 | .74 | .61 |
| AHS-R 5* | 0.8 | 0.6 | 1.6 | 5.1 | 15.1 | 19.2 | 57.7 | .75 | .65 |
| AHS-R 6* | 1.3 | 0.5 | 2.5 | 7.0 | 13.0 | 19.7 | 56.1 | .67 | .64 |
| AHS-R 7 | 1.3 | 0.6 | 3.0 | 8.4 | 20.0 | 20.9 | 45.8 | .64 | .59 |
| AHS-R 8* | 1.9 | 1.3 | 2.2 | 6.7 | 14.9 | 18.9 | 54.2 | .60 | .55 |
| AHS-R 9* | 1.9 | 0.8 | 2.7 | 5.1 | 13.9 | 20.0 | 55.6 | .62 | .54 |
| AHS-R 10* | 2.4 | 1.7 | 2.9 | 5.5 | 13.5 | 20.8 | 53.2 | .57 | .48 |
| AHS-R 11* | 4.1 | 2.5 | 4.6 | 7.8 | 19.2 | 17.1 | 44.7 | .50 | .43 |
| AHS-R 12* | 9.4 | 5.4 | 6.0 | 10.1 | 11.6 | 14.1 | 43.4 | .12 | .12 |
| AHS-R 13* | 2.1 | 2.5 | 4.8 | 8.2 | 11.1 | 19.5 | 51.8 | .44 | .42 |
| AHS-R 14 | 2.4 | 2.1 | 6.5 | 22.3 | 23.9 | 15.5 | 27.3 | .60 | .41 |
| AHS-R 15 | 2.9 | 3.6 | 7.6 | 17.3 | 21.7 | 16.5 | 30.4 | .64 | .40 |
| AHS-R 16 | 1.6 | 1.4 | 4.3 | 14.3 | 23.8 | 22.5 | 32.2 | .67 | .54 |
| AHS-R 17 | 1.0 | 2.1 | 4.3 | 14.3 | 22.3 | 21.1 | 35.0 | .67 | .53 |
| AHS-R 18 | 3.5 | 2.1 | 4.8 | 15.8 | 23.1 | 17.0 | 33.8 | .52 | .49 |
| AHS-R 19* | 17.6 | 11.7 | 21.2 | 18.1 | 11.4 | 8.6 | 11.4 | .51 | -.07 |
| AHS-R 20* | 8.4 | 7.9 | 12.5 | 22.7 | 14.3 | 12.4 | 21.9 | .67 | .18 |
| AHS-R 21* | 14.9 | 9.2 | 15.2 | 19.0 | 12.7 | 11.9 | 17.1 | .62 | .09 |
| AHS-R 22* | 11.1 | 7.1 | 15.4 | 22.3 | 12.8 | 13.9 | 17.3 | .71 | .14 |
| AHS-R 23* | 1.4 | 0.6 | 1.0 | 5.9 | 10.9 | 18.9 | 61.3 | .03 | .52 |
| AHS-R 24* | 18.7 | 16.6 | 29.0 | 20.8 | 8.1 | 2.9 | 4.0 | .41 | -.20 |
| AHS-R 25 | 13.2 | 11.4 | 26.5 | 28.4 | 10.5 | 4.4 | 5.7 | .52 | -.12 |
| AHS-R 26* | 3.0 | 2.1 | 6.7 | 17.7 | 19.3 | 21.2 | 30.0 | .69 | .42 |
| AHS-R 27* | 3.3 | 1.9 | 9.4 | 17.1 | 21.2 | 17.0 | 30.1 | .69 | .38 |
| AHS-R 28* | 8.9 | 3.8 | 7.0 | 15.8 | 18.1 | 15.5 | 30.9 | .53 | .31 |
| AHS-R 29* | 4.9 | 4.0 | 8.9 | 18.1 | 19.0 | 18.2 | 26.9 | .67 | .28 |
| AHS-R 30* | 1.9 | 1.3 | 5.7 | 14.7 | 19.7 | 23.5 | 33.3 | .50 | .46 |
| AHS-R 31* | 1.3 | 0.6 | 2.7 | 9.5 | 19.0 | 24.7 | 42.2 | .36 | .62 |
| AHS-R 32 | 1.7 | 0.6 | 2.1 | 8.6 | 18.1 | 23.8 | 45.2 | -.43 | -.59 |
| AHS-R 33 | 4.1 | 3.8 | 9.7 | 21.4 | 20.9 | 14.6 | 25.5 | .49 | .31 |

*Notes.* * indicates items from the original AHS.
