# Supplementary material for: The AHS-R: A holistic thinking measure with expanded theoretical domains and improved score reliability
Source: PLoS One. 2026 Jul 15;21(7):e0353378. doi: 10.1371/journal.pone.0353378 (PMC13372108; doi:10.1371/journal.pone.0353378)
Supplement: S2 Appendix — (DOCX) [file pone.0353378.s002.docx]

***Initial CFAs and Item Selection***

The second-order factor model with five dimensions (Model 1) resulted in poor fit ($X^{2}/df$ = 9.5, RMSEA = .112, CFI = 0.718, SRMR = 0.121). Most obvious issue was that the factor loading of Change on the second-order general factor was negative (standardized loading = -0.49, SE = 0.05, z-value = -12.46, p < .001). This finding was in line with the poor item-rest correlations of the of Change items (Table 1). An alternative second-order factor model (Model 2) with four first-order factors was fit. Global fit was improved but was still poor ($X^{2}/df$ = 7.4, RMSEA = .104, CFI = 0.819, SRMR = 0.076). In this solution, Item 32 (Sonradan fark ettiğimiz bir detay, bir olguya bakışımızı kökten değiştirebilir) showed negative factor loading and modification indices suggested it cross-loads with multiple first-order factors. Additionally, Item 12 (Choosing a middle ground in an argument should be avoided) showed low factor loadings (FL). We removed these four items and fitted another model (Model 3), which resulted in improved but not satisfactory fit ($X^{2}/df$ = 6.7, RMSEA = .099, CFI = 0.851, SRMR = 0.068). Model 4 excluded Item 31 (We should consider the situation a person is faced with, as well as his/her personality, in order to understand one’s behavior.), which showed multiple cross-loadings based on the modification indices from Model 3. Global fit improved ($X^{2}/df$ = 5.0, RMSEA = .089, CFI = 0.893, SRMR = 0.057) but Item 30 (It is not possible to understand the parts without considering the whole picture) identified as problematic due to cross-loadings, which was removed in Model 5. The global fit of Model 5 was not satisfactory (($X^{2}/df$ = 4.4, RMSEA = .073, CFI = 0.893, SRMR = 0.053).), and modification indices suggested removing Item 18 due to cross-loading with Causality and removing one of Causality 2 (Nothing is unrelated) or Causality 3 (Everything in the world is intertwined in a causal relationship.) due to correlated residuals which suggests one of the items may be redundant. We decided to remove Causality 2 because Causality 3 showed stronger factor loading. This model (Model 6) did not reach acceptable global fit values ($X^{2}/df$ = 3.6, RMSEA = .076, CFI = 0.927, SRMR = 0.048) and modification indices suggested removing Attitudes towards Contradiction 2 (Evrendeki her şey kendi içinde birbirine zıt özellikler barındırabilir) which showed cross-loadings and correlated residuals with other items. Model 7 applied these modifications but did not meet the criteria ($X^{2}/df$ = 3.3, RMSEA = .074, CFI = 0.933, SRMR = 0.045), and modification indices suggested removing Locus of Attention 8 (Bir sistem bozulduğunda tüm sisteme odaklanmak, bozuk parçaya odaklanmaktan daha gereklidir.) which showed cross-loadings. Model 8 showed good fit ($X^{2}/df$ = 3.0, RMSEA = .075, CFI = 0.938, SRMR = 0.041). In this model, Causality was measured with six indicators, Middle way five, Attitude towards contradiction three, Locus of attention four. To have a balanced number of items in each scale, we kept the best four items from Causality and Middle way subscales. This final model (Model 9) showed good fit to data ($X^{2}/df$ = 2.8, RMSEA = .071, CFI = 0.954, SRMR = 0.039)

Global goodness-of-fit of the second-order CFA models

|  | $X^{2}$ | *df* | $X^{2}$/*df* | RMSEA [90% CI] | CFI | SRMR |
| --- | --- | --- | --- | --- | --- | --- |
| Model 1 | 4663.022 | 490 | 9.5 | .112 [.108, .116] | 0.718 | 0.121 |
| Model 2 | 2173.569 | 295 | 7.4 | .104 [.099, .109] | 0.819 | 0.076 |
| Model 3 | 1653.784 | 248 | 6.7 | .099 [.094, .105] | 0.851 | 0.068 |
| Model 4 | 1130.482 | 226 | 5.0 | .089 [.084, .095] | 0.884 | 0.057 |
| Model 5 | 900.584 | 205 | 4.4 | .073 [.069, .078] | 0.893 | 0.053 |
| Model 6 | 600.015 | 166 | 3.6 | .076 [.069, .083] | 0.927 | 0.048 |
| Model 7 | 493.357 | 148 | 3.3 | .074 [.067, .082] | 0.933 | 0.045 |
| Model 8 | 398.639 | 131 | 3.0 | .075 [.067, .082] | 0.938 | 0.041 |
| Model 9 | 244.450 | 86 | 2.8 | .071 [.062, .081] | 0.954 | 0.039 |
