# Supplementary material for: The AHS-R: A holistic thinking measure with expanded theoretical domains and improved score reliability
Source: PLoS One. 2026 Jul 15;21(7):e0353378. doi: 10.1371/journal.pone.0353378 (PMC13372108; doi:10.1371/journal.pone.0353378)
Supplement: S3 Appendix — (DOCX) [file pone.0353378.s003.docx]

S3 Table. Global goodness-of-fit of the unidimensional IRT models before and after collapsing

|  |  |  | Global fit measures | | | | |
| --- | --- | --- | --- | --- | --- | --- | --- |
| Dimension | Model | Scale | $C_{2}$ | *df* | $C_{2}$/*df* | AIC | BIC |
| Causality | PCM | 7-point | 49.852 | 5 | 10.0 | 5196.570 | 5307.753 |
|  | PCM | 4-point | 48.375 | 5 | 9.7 | 3759.571 | 3817.386 |
|  | GPCM | 7-point | 24.020 | 2 | 12.0 | 5179.881 | 5304.406 |
|  | GPCM | 4-point | 27.223 | 2 | 13.6 | 3727.108 | 3798.264 |
|  |  |  |  |  |  |  |  |
| Midway | PCM | 7-point | 55.667 | 5 | 11.1 | 6353.178 | 6464.361 |
|  | PCM | 4-point | 34.137 | 5 | 6.8 | 4587.142 | 4644.957 |
|  | GPCM | 7-point | 17.202 | 2 | 8.6 | 6342.845 | 6467.370 |
|  | GPCM | 4-point | 16.268 | 2 | 8.1 | 4580.699 | 4651.856 |
|  |  |  |  |  |  |  |  |
| Contradiction | PCM | 7-point | 18.195 | 2 | 9.1 | 5647.624 | 5732.122 |
|  | PCM | 4-point | 15.198 | 2 | 7.6 | 3809.531 | 3854.004 |
|  | GPCM | 7-point | - | - | - | 5630.586 | 5723.980 |
|  | GPCM | 4-point | - | - | - | 3795.627 | 3848.995 |
|  |  |  |  |  |  |  |  |
| Attention | PCM | 7-point | 144.169 | 5 | 28.8 | 7947.535 | 8058.718 |
|  | PCM | 4-point | 114.671 | 5 | 22.9 | 5345.825 | 5403.640 |
|  | GPCM | 7-point | 25.214 | 2 | 12.6 | 7850.593 | 7975.118 |
|  | GPCM | 4-point | 18.090 | 2 | 9.0 | 5251.667 | 5322.824 |

*Notes.* PCM = Partial Credit Model. GPCM = Generalized Partial Credit Model.
