# Supplementary material for: The AHS-R: A holistic thinking measure with expanded theoretical domains and improved score reliability
Source: PLoS One. 2026 Jul 15;21(7):e0353378. doi: 10.1371/journal.pone.0353378 (PMC13372108; doi:10.1371/journal.pone.0353378)
Supplement: S4 Appendix — (DOCX) [file pone.0353378.s004.docx]

Item fit of the second-order factor GPCM with 4-point response scale

|  | ${S-X}^{2}$ | *df* | ${S-X}^{2}$*/df* | RMSEA |
| --- | --- | --- | --- | --- |
| AHS-R 1* | 60.95 | 35 | 1.74 | 0.03 |
| AHS-R 4* | 52.89 | 34 | 1.56 | 0.03 |
| AHS-R 5* | 70.43^a^ | 32 | 2.20 | 0.04 |
| AHS-R 6* | 53.63 | 33 | 1.63 | 0.03 |
| AHS-R 8* | 58.97 | 36 | 1.64 | 0.03 |
| AHS-R 9* | 46.55 | 35 | 1.33 | 0.02 |
| AHS-R 10* | 46.12 | 35 | 1.32 | 0.02 |
| AHS-R 11* | 75.78 | 45 | 1.68 | 0.03 |
| AHS-R 14 | 72.34 | 40 | 1.81 | 0.04 |
| AHS-R 16 | 64.49 | 36 | 1.79 | 0.04 |
| AHS-R 17 | 55.74 | 35 | 1.59 | 0.03 |
| AHS-R 26* | 47.60 | 36 | 1.32 | 0.02 |
| AHS-R 27* | 67.06 | 40 | 1.68 | 0.03 |
| AHS-R 28* | 116.75^a^ | 52 | 2.25 | 0.04 |
| AHS-R 29* | 91.10^a^ | 45 | 2.02 | 0.04 |

*items from original AHS

^a^ *p* < .001
