# Supplementary material for: The AHS-R: A holistic thinking measure with expanded theoretical domains and improved score reliability
Source: PLoS One. 2026 Jul 15;21(7):e0353378. doi: 10.1371/journal.pone.0353378 (PMC13372108; doi:10.1371/journal.pone.0353378)
Supplement: S5 Appendix — (DOCX) [file pone.0353378.s005.docx]

The bifactor model from which the hierarchical omega coefficients were estimated.

AHS-R 1

**0.4**

**0.4**

**0.7**

AHS-R 4 Causality

**0.5**

**0.7**

AHS-R 5

**0.4**

**0.8**

AHS-R 6

**0.4**

**0.8**

AHS-R 8

**0.5**

**0.7**

AHS-R 9 Midway

**0.5**

**0.7**

AHS-R 10

**0.5**

**0.6**

**0.5**

Holism AHS-R 11

**0.5**

AHS-R 14

**0.5**

**0.5**

**0.7**

AHS-R 16 Contradiction

**0.4**

**0.7**

AHS-R 17

**0.5**

AHS-R 26

**0.7**

**0.4**

AHS-R 27

**0.8**

**0.5**

**0.4**

AHS-R 28 Attention

**0.7**

**0.4**

AHS-R 29
