# Supplementary material for: The AHS-R: A holistic thinking measure with expanded theoretical domains and improved score reliability
Source: PLoS One. 2026 Jul 15;21(7):e0353378. doi: 10.1371/journal.pone.0353378 (PMC13372108; doi:10.1371/journal.pone.0353378)
Supplement: S6 Appendix — (DOCX) [file pone.0353378.s006.docx]

**Summary of Prior Psychometric Work on the AHS**

|  |  | **Causality** | | | | | | **Midway** | | | | | | **Contradiction** | | | | | | **Change** | | | | | | **Attention** | | | | | |
| --- | --- | --- | --- | --- | --- | --- | --- | --- | --- | --- | --- | --- | --- | --- | --- | --- | --- | --- | --- | --- | --- | --- | --- | --- | --- | --- | --- | --- | --- | --- | --- |
| **Studies** | | **C** | **L** | **M** | **S1** | **S2** | **R** | **C** | **L** | **M** | **S1** | **S2** | **R** | **C** | **L** | **M** | **S1** | **S2** | **R** | **C** | **L** | **M** | **S1** | **S2** | **R** | **C** | **L** | **M** | **S1** | **S2** | **R** |
| **Choi** | **Sencan** |  |  |  |  |  |  |  |  |  |  |  |  |  |  |  |  |  |  |  |  |  |  |  |  |  |  |  |  |  |  |
| **1** | **1** | **X** | **X** | **X** | **X** | **X** | **X** |  |  |  |  |  |  |  |  |  |  |  |  |  |  |  |  |  |  |  |  |  |  |  |  |
| **2** | **2** | **X** | **X** |  | **X** |  |  |  |  |  |  |  |  |  |  |  |  |  |  |  |  |  |  |  |  |  |  |  |  |  |  |
| **3** | **3** | **X** | **X** | **X** | **X** |  |  |  |  |  |  |  |  |  |  |  |  |  |  |  |  |  |  |  |  |  |  |  |  |  |  |
| **4** | **4** | **X** | **X** | **X** | **X** | **X** | **X** |  |  |  |  |  |  |  |  |  |  |  |  |  |  |  |  |  |  |  |  |  |  |  |  |
| **5** | **5** | **X** | **X** |  | **X** | **X** | **X** |  |  |  |  |  |  |  |  |  |  |  |  |  |  |  |  |  |  |  |  |  |  |  |  |
| **6** | **6** | **X** | **X** |  | **X** | **X** | **X** |  |  |  |  |  |  |  |  |  |  |  |  |  |  |  |  |  |  |  |  |  |  |  |  |
|  | **7** |  |  |  | **X** |  |  |  |  |  |  |  |  |  |  |  |  |  |  |  |  |  |  |  |  |  |  |  |  |  |  |
| **7** | **8** |  |  |  |  |  |  | **X** | **X** | **X** | **X** | **X** | **X** |  |  |  |  |  |  |  |  |  |  |  |  |  |  |  |  |  |  |
| **8** | **9** |  |  |  |  |  |  | **X** | **X** |  | **X** | **X** | **X** |  |  |  |  |  |  |  |  |  |  |  |  |  |  |  |  |  |  |
| **9** | **10** |  |  |  |  |  |  | **X** | **X** | **X** | **X** | **X** | **X** |  |  |  |  |  |  |  |  |  |  |  |  |  |  |  |  |  |  |
| **10** | **11** |  |  |  |  |  |  | **X** | **X** |  | **X** | **X** | **X** |  |  |  |  |  |  |  |  |  |  |  |  |  |  |  |  |  |  |
| **11** | **12** |  |  |  |  |  |  | **X** |  |  | **X** |  |  |  |  |  |  |  |  |  |  |  |  |  |  |  |  |  |  |  |  |
| **12** | **13** |  |  |  |  |  |  | **X** | **X** | **X** | **X** |  |  |  |  |  |  |  |  |  |  |  |  |  |  |  |  |  |  |  |  |
|  | **14** |  |  |  |  |  |  |  |  |  |  |  |  |  |  |  | **X** | **X** | **X** |  |  |  |  |  |  |  |  |  |  |  |  |
|  | **15** |  |  |  |  |  |  |  |  |  |  |  |  |  |  |  | **X** |  |  |  |  |  |  |  |  |  |  |  |  |  |  |
|  | **16** |  |  |  |  |  |  |  |  |  |  |  |  |  |  |  | **X** | **X** | **X** |  |  |  |  |  |  |  |  |  |  |  |  |
|  | **17** |  |  |  |  |  |  |  |  |  |  |  |  |  |  |  | **X** | **X** | **X** |  |  |  |  |  |  |  |  |  |  |  |  |
|  | **18** |  |  |  |  |  |  |  |  |  |  |  |  |  |  |  | **X** |  |  |  |  |  |  |  |  |  |  |  |  |  |  |
| **13** | **19** |  |  |  |  |  |  |  |  |  |  |  |  |  |  |  |  |  |  | **X** | **X** | **X** | **X** |  |  |  |  |  |  |  |  |
| **14** | **20** |  |  |  |  |  |  |  |  |  |  |  |  |  |  |  |  |  |  | **X** | **X** | **X** | **X** |  |  |  |  |  |  |  |  |
| **15** | **21** |  |  |  |  |  |  |  |  |  |  |  |  |  |  |  |  |  |  | **X** | **X** |  | **X** |  |  |  |  |  |  |  |  |
| **16** | **22** |  |  |  |  |  |  |  |  |  |  |  |  |  |  |  |  |  |  | **X** | **X** |  | **X** |  |  |  |  |  |  |  |  |
| **17** | **23** |  |  |  |  |  |  |  |  |  |  |  |  |  |  |  |  |  |  | **X** |  |  | **X** |  |  |  |  |  |  |  |  |
| **18** | **24** |  |  |  |  |  |  |  |  |  |  |  |  |  |  |  |  |  |  | **X** | **X** | **X** | **X** |  |  |  |  |  |  |  |  |
|  | **25** |  |  |  |  |  |  |  |  |  |  |  |  |  |  |  |  |  |  |  |  |  | **X** |  |  |  |  |  |  |  |  |
| **19** | **26** |  |  |  |  |  |  |  |  |  |  |  |  |  |  |  |  |  |  |  |  |  |  |  |  | **X** | **X** | **X** | **X** | **X** | **X** |
| **20** | **27** |  |  |  |  |  |  |  |  |  |  |  |  |  |  |  |  |  |  |  |  |  |  |  |  | **X** | **X** | **X** | **X** | **X** | **X** |
| **21** | **28** |  |  |  |  |  |  |  |  |  |  |  |  |  |  |  |  |  |  |  |  |  |  |  |  | **X** | **X** |  | **X** | **X** | **X** |
| **22** | **29** |  |  |  |  |  |  |  |  |  |  |  |  |  |  |  |  |  |  |  |  |  |  |  |  | **X** | **X** | **X** | **X** | **X** | **X** |
| **23** | **30** |  |  |  |  |  |  |  |  |  |  |  |  |  |  |  |  |  |  |  |  |  |  |  |  | **X** |  |  | **X** |  |  |
| **24** | **31** |  |  |  |  |  |  |  |  |  |  |  |  |  |  |  |  |  |  |  |  |  |  |  |  | **X** |  |  | **X** |  |  |
|  | **32** |  |  |  |  |  |  |  |  |  |  |  |  |  |  |  |  |  |  |  |  |  |  |  |  |  |  |  | **X** |  |  |
|  | **33** |  |  |  |  |  |  |  |  |  |  |  |  |  |  |  |  |  |  |  |  |  |  |  |  |  |  |  | **X** |  |  |
|  |  | **Causality** | | | | | | **Midway** | | | | | | **Contradiction** | | | | | | **Change** | | | | | | **Attention** | | | | | |
| **Factor**  **Correlations** | **1** | **1** | **1** | **1** | **1** | **1** | **1** |  |  |  |  |  |  |  |  |  |  |  |  |  |  |  |  |  |  |  |  |  |  |  |  |
|  | **2** | **.25** | **.36** | **.49** | **.47** | **-** | **-** | **1** | **1** | **1** | **1** | **1** | **1** |  |  |  |  |  |  |  |  |  |  |  |  |  |  |  |  |  |  |
|  | **3** | **-** | **-** | **-** | **.81** | **-** | **-** | **-** | **-** | **-** | **.39** | **-** | **-** | **-** | **-** | **-** | **1** | **1** | **1** |  |  |  |  |  |  |  |  |  |  |  |  |
|  | **4** | **.07** | **.17** | **.38** | **.24** | **-** | **-** | **.15** | **.21** | **.40** | **-.04** | **-** | **-** | **-** | **-** | **-** | **.26** | **-** | **-** | **1** | **1** | **1** | **1** | **-** | **-** |  |  |  |  |  |  |
|  | **5** | **.23** | **.37** | **.40** | **.11** | **-** | **-** | **.19** | **.30** | **.42** | **.33** | **-** | **-** | **-** | **-** | **-** | **.33** | **-** | **-** | **.13** | **.12** | **.71** | **.19** | **-** | **-** | **1** | **1** | **1** | **1** | **1** | **1** |
| Reliability | **α** | **.76** | **.71** | **.79** | **.86** | **.87** | **.86** | **.71** | **.62** | **.68** | **.74** | **.79** | **.80** | **-** | **-** | **-** | **.67** | **.76** | **.77** | **.71** | **.56** | **.77** | **.69** | **-** | **-** | **.67** | **.68** | **.82** | **.66** | **.82** | **.83** |
|  | **ω** | **-** | **-** | **.80** | **-** | **-** | **.93** | **-** | **-** | **.73** | **-** | **-** | **.87** | **-** | **-** | **-** | **-** | **-** | **.83** | **-** | **-** | **.76** | **-** | **-** | **-** | **-** | **-** | **.81** | **-** | **-** | **.88** |

*Notes.* C = Choi et al. (2007), L = Lechuga et al., (2011); M = Martin –Fernandez et al., (2022), S1 = Study 1; S2 = Model 9 in Study 2, and R = Final Revised-AHS in Study 2

**Original and newly developed AHS items tested in prior and current psychometric studies**

| **Analysis-Holism Scale (Choi et al., 2007)** | **Modified AHS-33 Used in Study 1 (English)**  **(Şencan et al., 2021)** | **Modified AHS-33 Used in Study 1 (Turkish)**  **(Şencan et al., 2021)** |
| --- | --- | --- |
| **Causality** | **Causality** | **Nedensellik** |
| 1. Everything in the universe is somehow related to each other. ^L, M, M12^ | 1. Everything in the universe is somehow related to each other. ^S2,^ ^R^ | 1. Evrendeki her şey bir şekilde birbiriyle ilişkilidir.  ^S2, R^ |
| 2. Nothing is unrelated.  ^L, M^ | 2. Nothing is unrelated. ^S2^ | 2. Aralarında ilişki olmayan hiçbir şey yoktur.  ^S2^ |
| 3. Everything in the world is intertwined in a causal relationship.  ^L, M^ | 3. Everything in the world is intertwined in a causal relationship.  ^S2^ | 3. Dünyadaki her şey nedensel bir ilişkiyle iç içe geçmiştir.  ^S2^ |
| 4. Even a small change in any element of the universe can lead to significant alterations in other elements.  ^L, M, M21^ | 4. Even a small change in any element of the universe can lead to significant alterations in other elements.  ^S2, R^ | 4. Evrenin herhangi bir unsurundaki ufak bir değişiklik bile öteki unsurlarda önemli değişimlere yol açabilir. ^S2,^ ^R^ |
| 5. Any phenomenon has numerous numbers of causes, although some of the causes are not known.  ^L, M, M12^ | 5. Any phenomenon has numerous numbers of causes, although some of the causes are not known.  ^S2, R^ | 5. Her olayın çok sayıda nedeni vardır, her ne kadar bazıları bilinmese de.  ^S2, R^ |
| 6. Any phenomenon entails a numerous number of consequences, although some of them may not be known.  ^L, M^ | 6. Any phenomenon entails a numerous number of consequences, although some of them may not be known.  ^S2, R^ | 6. Her olay sayısız sonuç doğurur, her ne kadar bazıları bilinmese de.  ^S2, R^ |
|  | 7. It is possible that there is a causal relationship between two events that seem unrelated at first sight. ^S2, S1^ | 7. İlk bakışta ilişkisiz gibi görünen iki olayın birbirine bir nedenler zinciriyle bağlı olması muhtemeldir. ^S2, S1^ |
| **Attitude Toward Contradictions** | **Middle Way Approach** | **Orta Yolculuk** |
| 7. It is more desirable to take the middle ground than go to extremes.  ^L, M, M12^ | 8. It is more desirable to take the middle ground than go to extremes.  ^S2, R^ | 8. Aşırıya kaçmaktansa orta yolu bulmak daha caziptir.  ^S2, R^ |
| 8. When disagreement exists among people, they should search for ways to compromise and embrace everyone’s opinions.  ^L, M^ | 9. When disagreement exists among people, they should search for ways to compromise and embrace everyone’s opinions.  ^S2, R^ | 9. İnsanlar aralarında anlaşmazlık olduğunda, uzlaşmak ve herkesin fikrine kucak açmak için yollar aramalıdırlar.  ^S2, R^ |
| 9. It is more important to find a point of compromise than to debate who is right/wrong, when one’s opinions conflict with other’s opinions.  ^L, M, M12^ | 10. It is more important to find a point of compromise than to debate who is right/wrong, when one’s opinions conflict with other’s opinions.  ^S2, R^ | 10. Birinin görüşleri bir başkasının görüşleriyle ters düştüğünde uzlaşma noktası bulmak, kimin haklı/haksız olduğunu tartışmaktan daha önemlidir.  ^S2, R^ |
| 10. It is desirable to be in harmony, rather than in discord, with others of different opinions than one’s own.  ^L, M^ | 11. It is desirable to be in harmony, rather than in discord, with others of different opinions than one’s own.  ^S2, R^ | 11. Kendinden farklı görüştekilerle anlaşmazlık içinde olmaktansa, uyum içinde olmak daha caziptir.  ^S2, R^ |
| 11. Choosing a middle ground in an argument should be avoided. ^*, L, M^ | 12. Choosing a middle ground in an argument should be avoided. ^*, S2^ | 12. Bir tartışmada orta yolu seçmekten kaçınılmalıdır.  ^*, S2^ |
| 12. We should avoid going to extremes.  ^L, M, M12^ | 13. We should avoid going to extremes. ^S2^ | 13. Aşırıya kaçmaktan kaçınmalıyız.  ^S2^ |
|  | **Attitude Toward Contradictions (Tolerance of Contradictions)** | **Çelişkiye Dair Tutum (Çelişkiye Tahammül)** |
|  | 14. A lot of issues are nuanced enough to contain contradictory truths simultaneously. ^S1, S2, R^ | 14. Birçok mesele kendi içinde zıtlıklar içerecek kadar karmaşıktır. ^S1, S2, R^ |
|  | 15. Everything in the universe can contain incompatible characteristics. ^S1, S2^ | 15. Evrendeki her şey kendi içinde birbirine zıt özellikler barındırabilir. ^S1, S2^ |
|  | 16. Having a particular characteristic does not prevent having also an opposing feature. ^S1, S2, R^ | 16. Bir şeyin belli bir özellikle nitelendirilmesi, karşıt bir özelliğe de sahip olmasına engel değildir. ^S1, S2, R^ |
|  | 17. A phenomenon which looks positive usually contains negativity in it as well. ^S1, S2, R^ | 17. Çok olumlu gözüken bir olgu veya kavram, çoğu zaman içinde olumsuzu da barındırır. ^S1, S2, R^ |
|  | 18. Most of our adverse experiences carry the seeds of future positive influences. ^S1, S2^ | 18. Başımıza gelen olumsuz olayların çoğu aslında ileride doğacak olumlu etkilerin de tohumunu içinde taşır. ^S1, S2^ |
| **Perception of Change** | **Perception of Change** | **Değişim Beklentisi** |
| 13. Every phenomenon in the world moves in predictable directions. ^*, L M, M12^ | 19. Every phenomenon in the world moves in predictable directions. ^*^ | 19. Dünyadaki bütün olaylar öngörülebilir doğrultuda gelişir. ^*^ |
| 14. A person who is currently living a successful life will continue to stay successful. ^*, L, M, M12^ | 20. A person who is currently living a successful life will continue to stay successful. ^*^ | 20. Şu anda başarılı bir hayat yaşayan bir insan, başarılı olmaya devam edecektir. ^*^ |
| 15. An individual who is currently honest will stay honest in the future. ^*, L, M^ | 21. An individual who is currently honest will stay honest in the future. ^*^ | 21. Şu anda dürüst olan bir kişi gelecekte dürüst kalacaktır. ^*^ |
| 16. If an event is moving toward a certain direction, it will continue to move toward that direction. ^*, L, M^ | 22. If an event is moving toward a certain direction, it will continue to move toward that direction. ^*^ | 22. Eğer bir olay belli bir yönde gelişiyorsa, o yönde gitmeye devam edecektir. ^*^ |
| 17. Current situations can change at any time.  ^L, M^ | 23. Current situations can change at any time. | 23. Mevcut durumlar her an değişebilir. |
| 18. Future events are predictable based on present situations. ^*, L, M, M12^ | 24. Future events are predictable based on present situations. ^*^ | 24. Gelecek olaylar mevcut durumlara dayanarak öngörülebilir. ^*^ |
|  | 25. A continuously increasing state is more likely to increase rather than to decrease. ^*, S1^ | 25. Yükselmekte olan bir şeyin yükselmeye devam etmesi, düşmeye başlamasından daha olasıdır. ^*, S1^ |
| **Locus of Attention** | **Locus of Attention** | **Dikkat Odağı** |
| 19. The whole, rather than its parts, should be considered in order to understand a phenomenon.  ^L, M, M12^ | 26. The whole, rather than its parts, should be considered in order to understand a phenomenon.  ^S2, R^ | 26. Bir olguyu anlamak için parçalarındansa bütünü göz önüne alınmalıdır.  ^S2, R^ |
| 20. It is more important to pay attention to the whole than its parts.  ^L, M, M12^ | 27. It is more important to pay attention to the whole than its parts.  ^S2, R^ | 27. Parçalardansa bütüne dikkat etmek daha önemlidir. ^S2, R^ |
| 21. The whole is greater than the sum of its parts. ^L, M^ | 28. The whole is greater than the sum of its parts.  ^S2, R^ | 28. Bütün, parçalarının toplamından daha büyüktür.  ^S2, R^ |
| 22. It is more important to pay attention to the whole context rather than the details.  ^L, M, M12^ | 29. It is more important to pay attention to the whole context rather than the details.  ^S2, R^ | 29. Ayrıntılardansa olayın bütününe dikkat etmek daha önemlidir.  ^S2, R^ |
| 23. It is not possible to understand the parts without considering the whole picture.  ^L, M^ | 30. It is not possible to understand the parts without considering the whole picture.  ^S2^ | 30. Büyük resmi hesaba katmadan parçaları anlamak mümkün değildir.  ^S2^ |
| 24. We should consider the situation a person is faced with, as well as his/her personality, in order to understand one’s behavior.  ^L, M^ | 31. We should consider the situation a person is faced with, as well as his/her personality, in order to understand one’s behavior. | 31. Birinin davranışını anlamak için kişiliğinin yanı sıra karşı karşıya kaldığı durumu da göz önünde bulundurmalıyız. |
|  | 32. A detail noticed afterwards can totally change our view of a phenomenon. ^*, S1, S2^ | 32. Sonradan fark ettiğimiz bir detay, bir olguya bakışımızı kökten değiştirebilir.  ^*, S1, S2^ |
|  | 33. When a system corrupted, it is more important to focus on the whole system rather than on the single broken part. ^S1, S2^ | 33. Bir sistem bozulduğunda tüm sisteme odaklanmak, bozuk parçaya odaklanmaktan daha gereklidir. ^S1, S2^ |
| *Notes*. Blank cells mean that corresponding items does not exist in the Choi et al.’s AHS.  ^S1^ Reflects items generated by Şencan et al. (2021) in Study 1.  ^S2^ Reflects items in Model 9 in Study 2.  ^R^ Reflects Final AHS-R items.  ^*^ Reflects reverse coded items.  ^L^ Reflects items used in Lechuga et al. (2011).  ^M^ Reflects items used in Martin-Fernandez et al. (2022).  ^M12^ Reflects AHS-12 items adapted by Martin-Fernandez et al. (2022). | | |
|  | | |
