# Supplementary material for: The AHS-R: A holistic thinking measure with expanded theoretical domains and improved score reliability
Source: PLoS One. 2026 Jul 15;21(7):e0353378. doi: 10.1371/journal.pone.0353378 (PMC13372108; doi:10.1371/journal.pone.0353378)
Supplement: S7 Appendix — (DOCX) [file pone.0353378.s007.docx]

**The Analysis-Holism Revised Scale (AHS-R) English Items**

| **Causality** |
| --- |
| 1. Everything in the universe is somehow related to each other. |
| 2. Even a small change in any element of the universe can lead to significant alterations in other elements. |
| 3. Any phenomenon has numerous numbers of causes, although some of the causes are not known. |
| 4. Any phenomenon entails a numerous number of consequences, although some of them may not be known. |
| **Middle Way** |
| 5. It is more desirable to take the middle ground than go to extremes. |
| 6. When disagreement exists among people, they should search for ways to compromise and embrace everyone’s opinions. |
| 7. It is more important to find a point of compromise than to debate who is right/wrong, when one’s opinions conflict with other’s opinions. |
| 8. It is desirable to be in harmony, rather than in discord, with others of different opinions than one’s own. |
| **Attitude Toward Contradiction** |
| 9. A lot of issues are nuanced enough to contain contradictory truths simultaneously. |
| 10. Having a particular characteristic does not prevent having also an opposing feature. |
| 11. A phenomenon which looks positive usually contains negativity in it as well. |
| **Locus of Attention** |
| 12. The whole, rather than its parts, should be considered in order to understand a phenomenon. |
| 13. It is more important to pay attention to the whole than its parts. |
| 14. The whole is greater than the sum of its parts. |
| 15. It is more important to pay attention to the whole context rather than the details. |

**The Analysis-Holism Revised Scale (AHS-R) Turkish Items**

| **Nedensellik** |
| --- |
| 1. Evrendeki her şey bir şekilde birbiriyle ilişkilidir. |
| 2. Evrenin herhangi bir unsurundaki ufak bir değişiklik bile öteki unsurlarda önemli değişimlere yol açabilir. |
| 3. Her olayın çok sayıda nedeni vardır, her ne kadar bazıları bilinmese de. |
| 4. Her olay sayısız sonuç doğurur, her ne kadar bazıları bilinmese de. |
| **Orta Yolculuk** |
| 5. Aşırıya kaçmaktansa orta yolu bulmak daha caziptir. |
| 6. İnsanlar aralarında anlaşmazlık olduğunda, uzlaşmak ve herkesin fikrine kucak açmak için yollar aramalıdırlar. |
| 7. Birinin görüşleri bir başkasının görüşleriyle ters düştüğünde uzlaşma noktası bulmak, kimin haklı/haksız olduğunu tartışmaktan daha önemlidir. |
| 8. Kendinden farklı görüştekilerle anlaşmazlık içinde olmaktansa, uyum içinde olmak daha caziptir. |
| **Çelişkiye Dair Tutum** |
| 9. Birçok mesele kendi içinde zıtlıklar içerecek kadar karmaşıktır. |
| 10. Bir şeyin belli bir özellikle nitelendirilmesi, karşıt bir özelliğe de sahip olmasına engel değildir. |
| 11. Çok olumlu gözüken bir olgu veya kavram, çoğu zaman içinde olumsuzu da barındırır. |
| **Dikkat Odağı** |
| 12. Bir olguyu anlamak için parçalarındansa bütünü göz önüne alınmalıdır. |
| 13. Parçalardansa bütüne dikkat etmek daha önemlidir. |
| 14. Bütün, parçalarının toplamından daha büyüktür. |
| 15. Ayrıntılardansa olayın bütününe dikkat etmek daha önemlidir. |
